# Transcriptomic profiling for prolonged drought in *Dendrobium catenatum*

**DOI:** 10.1038/sdata.2018.233

**Published:** 2018-10-30

**Authors:** Xiao Wan, Long-Hai Zou, Bao-Qiang Zheng, Ying-Qiu Tian, Yan Wang

**Affiliations:** 1 State Key Laboratory of Tree Genetics and Breeding, Key Laboratory of Tree Breeding and Cultivation of State Forestry Administration, Research Institute of Forestry, Chinese Academy of Forestry, Beijing 100091, China; 2 Wenshan Academy of Agriculture Sciences, NO. 2 in Taikang Road (West), Wenshan 663099, China

**Keywords:** Drought, Transcriptomics, RNA sequencing

## Abstract

Orchid epiphytes, a group containing at least 18,000 species, thrive in habitats that often undergo periodic drought stress. However, few global gene expression profiling datasets have been published for studies addressing the drought-resistant mechanism of this special population. In this study, an experiment involving the effect of continuous drought treatments on an epiphytic orchid, *Dendrobium catenatum*, was designed to generate 39 mature-leaf-tissue RNA-seq sequencing datasets with over two billion reads. These datasets were validated by a series of quality assessments including RNA sample quality, RNA-seq read quality, and global gene expression profiling. We believe that these comprehensive transcriptomic resources will allow a better understanding of the drought-resistant mechanisms of orchid epiphytes.

## Background & Summary

In response to prolonged water deficit stress, plants have evolved coping mechanisms to increase their drought tolerance through physical adaptations, molecular regulations, and environmentally suitable metabolic pathways^1–3^. Most studies concerning drought stress mechanisms have been performed in *Arabidopsis thaliana* and other drought-intolerant C_3_ plants^2^. Studying a highly resistant plant that has been shaped by natural selection is the most direct and effective way to extract crucial genes and determine the main metabolic pathways of the drought stress procedure.

In the wild, most epiphytic orchids, a prosperous group containing over 18,000 species, take root on the surface of tree bark or rocks^4,5^. Due to the poor moisture supply in these habitats^6^, these plants usually suffer periodic water shortage^7^. While adapting to harsh habitats, some orchid species have evolved succulent storage-organs, such as pseudobulbs^8,9^, thick leaves^10^, and crassulacean acid metabolism (CAM)^11^, a photosynthetic pathway with high water-use efficiency^12^. Morphological and anatomical studies show that orchid plants possess desirable qualities for mitigating drought stress^10,13,14^. By measuring physiological indexes and secondary metabolites of *Dendrobium moniliforme*^15^, Wu *et al.* found that increasing antioxidant enzyme activities and osmolytes play an important role in protecting plants under drought stress. Although several physiological traits might provide clues for the mechanism of drought resistance, there is no large data set that allows holistic understanding. Unfortunately, few comprehensive transcriptomic profiling studies that address drought resistance have been published.

Comparing the two published genomes from epiphytic orchid species^16,17^, *Phalaenopsis equestris* and *Dendrobium catenatum*, the latter possesses more Heat-shock protein 70 family members and R genes^17^, which suggests that *D. catenatum* can tolerate a much wider variety of environments and has superior qualities for adverse resistance. A previous study demonstrates that *D. catenatum* uses the facultative CAM pathway as a drought-enduring process^11^. Hence, this species can be considered as drought-resistant material useful for elucidating mechanisms of mitigating drought stress in epiphytic orchids. Previous studies show that the circadian clock modifies responsiveness to environmental input and stress according to the time of day^18–21^. With regard to the correlation between CAM and circadian rhythm^22^. the conventional sampling tactics that focus on a single time point per day should be abandoned as, if the daylight sampling time is fixed, some important clues to key resistance genes could be missed.

**Figure 1:**
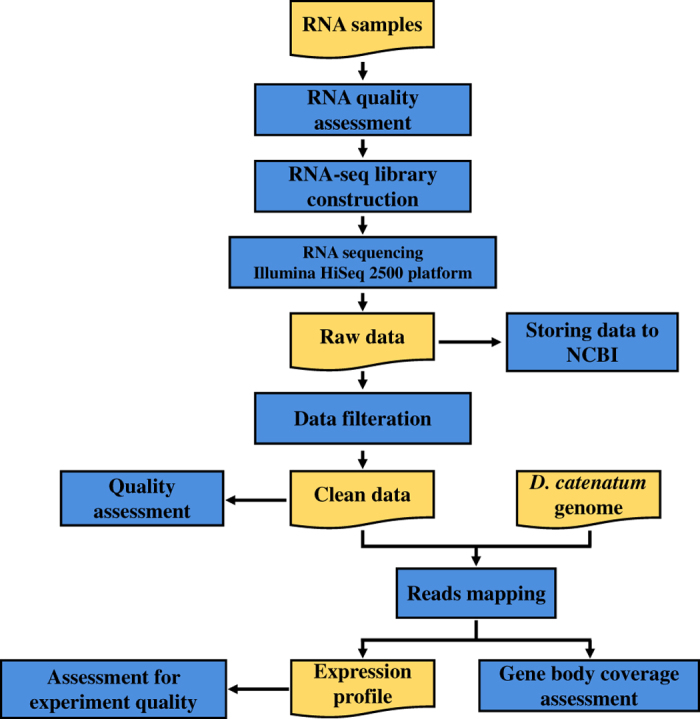
Overview of the experimental design and analysis pipeline. The raw data were filtered using the package Fastq_clean, and clean data were assessed using FastQC and MultiQC. The clean reads were mapped to the *D. catenatum* genome (GenBank Assembely ID ASM160598v2) using Hisat2. The package ReSQC was used to calculate RNA-seq reads coverage over the gene body. Gene abundance was quantified using DESeq2.

In the current study, *D. catenatum* plants were subjected to continuous drought treatments by simulating their natural environment under controlled conditions. Sampling time points were set for both day and night during the drought procedure. A dataset containing 39 RNA-seq with over 41 million sequence reads per sample was generated using the Illumina HiSeq 2500 platform. We assessed RNA sample quality, RNA-seq read quality, and the global gene profile (Fig. 1) to ensure the dependability of our dataset. We believe that these transcriptomic profiles will contribute to a comprehensive understanding of the mechanism of drought resistance in *D. catenatum*.

## Methods

### Plant material and experimental design

Clones of *D. catenatum* were planted in transparent plastic pots (5.0 cm in diameter) with sphagnum moss as the matrix. Eight-month-old plants were transferred into a phytotron chamber (12/12 h light/dark, light intensity ~100 μmol m^−2^s^−1^; 28/22 °C day/night; and relative humidity 60/70% day/night) and adapted to the controlled conditions for 10 days before being used for the follow-up experiment. The experiments were conducted on initially healthy individuals (~12 cm height). Plants were irrigated on the first day and then water was withheld to mimic drought stress. We collected leaf samples when the volumetric water content of the base material declined to ~30–35%, ~10–15, and ~0%, respectively, at both 09:00 h and 21:00 h (Fig. 1). The fourth and fifth leaves (mature leaf) from the apex of each plant were harvested and mixed to create one sample. These samples were immediately frozen in liquid N_2_ and stored at −80 °C.

### RNA isolation and sequencing

Total RNA was extracted from the samples mentioned above (Table 1) using the RNAprep Pure Plant Kit (No. DP441; Polysaccharides & Polyphenolics-rich; Tiangen Co. Ltd, Beijing, China; http://www.tiangen.com/) according to the manufacturer’s protocols. RNA purity was estimated using a NanoPhotometer^®^ spectrophotometer (Implen, CA, USA). RNA quality was assessed using an RNA Nano 6000 Assay Kit of the Bioanalyzer 2100 system (Agilent Technologies, CA, USA). RNA samples of acceptable quality were used to construct non-strand-specific sequencing libraries with the TruSeq RNA Sample Prep Kit (Illumina, CA, USA). These libraries were sequenced using the PE150 mode on an Illumina HiSeq2500 platform at Novogene Corporation (Beijing, China; http://www.novogene.com/).

### Data filtering and assessment

The raw data (raw reads; Data Citation 1) were filtered using Fastq_clean v2.0^23^. Sequencing adapters, low-quality bases, viral sequences, and rRNA sequences were cleaned. The criteria for this filtering procedure were set as follows: (1) RNA 5′ and 3′ adapters were set as [5′-AATGATACGGCGACCACCGAGATCTACACTCTTTCCCTACACGACGCTCTTCCGATCT-3′] and [5′-GATCGGAAGAGCACACGTCTGAACTCCAGTCAC (index) ATCTCGTATGCCGTCTTCTGCTTG-3’] (the indexes are listed in Table 1), respectively; (2) bases with a phred quality score below 20 were clipped from both ends of reads; (3) after low-quality bases were trimmed, reads containing over two “N” were discarded; (4) reads with a length shorter than 75 nt were discarded; and (5) the parameters for BWA v0.5.7^24^ were set as recommended according to Fastq_clean instructions. The statistics of clean reads are listed in Table 1. The quality of the clean data was evaluated using the package FastQC v0.11.7 (http://www.bioinformatics.babraham.ac.uk/projects/fastqc/) and then summarized using MultiQC v1.3^25^.

### Gene quantification and detection of read coverage skewness

The clean reads were mapped to the *D. catenatum* genome^17^ (GenBank Assembly ID ASM160598v2) using Hisat2^26^ with default parameters. Salmon v0.9.1^27^ was used to estimate gene abundance as read counts in the alignment-based mode. The raw read counts were imported into the R package DESeq2^28^ for normalization. We used the package ReSQC^29^ to assess RNA-seq read coverage skewness over the gene body based on the above mapping results.

### Assessment of sample composition

A heatmap for cluster relationships among samples representing Poisson distance were generated with raw read counts. The R package PoiClaClu^30^ was used for the calculation of Poisson distance, and the R package Pheatmap (https://cran.r-project.org/web/packages/pheatmap/index.html) for visualization. A principal component analysis (PCA) was also employed to assess sample relationships based on rlog-transformed values of raw read counts.

### Gene hierarchical clustering and Gene Ontology (GO) analysis

**Figure 2:**
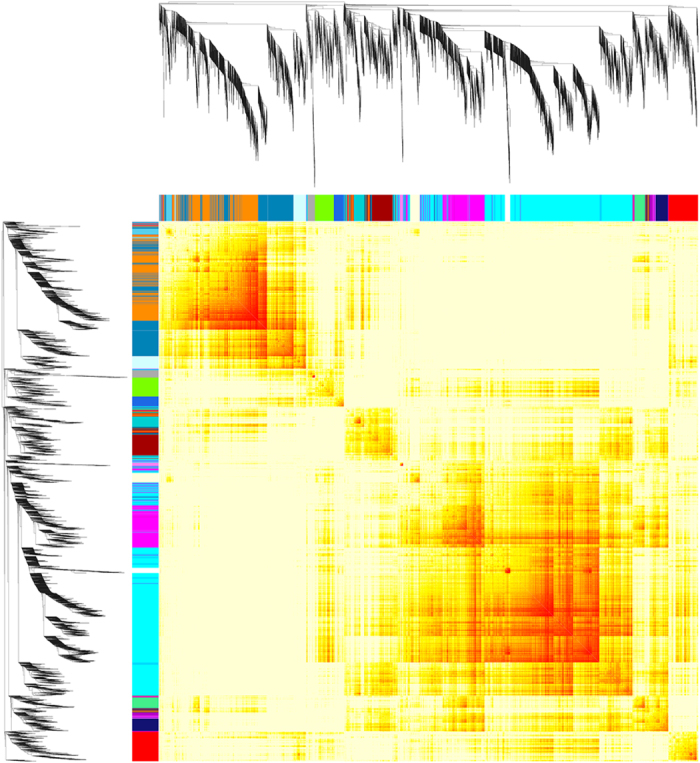
Topological overlap matrix plot. Seventeen color-coded modules were detected and Branches in the hierarchical clustering dendrograms correspond to modules (clusters).

To determine the highly correlated genes in this prolonged drought experiment, weighted gene co-expression network analysis (WGCNA)^31^ was used to detected gene clusters (modules) on normalized read counts (Data Citation 2) using the WGCNA v1.63^32,33^ package in R. This analysis generated a topological overlap matrix plot (Fig. 2) that illustrated the relationships among gene clusters. To give an insight into the functions of both genes and gene clusters, we performed GO enrichment analysis using Gogsea, a web tool from Omicshare (http://www.omicshare.com/tools/Home/Soft/gogsea). The edge information of each gene cluster and the results of both GO annotation and GO enrichment are stored in the Figshare repository (Data Citation 3).

### Code availability

The R scripts for reads count filtration and normalization, heatmap illustration, PCA and WGCNA are available in Figshare (Data Citation 4).

## Data Records

The RNA-seq raw data of 39 samples are deposited at the NCBI Sequence Read Archive (Data Citation 1).

Supplementary materials are available on the Figshare data management platform (Data Citations 2, 3, 4). Data Citation 2 provides expression profiles of raw read counts and normalized read counts; Data Citation 3 contains WGCNA results, GO annotation for all genes, and GO enrichment for gene clusters. Data Citation 4 is dedicated to the R scripts in this study.

## Technical Validation

### RNA quality control

The quality of total RNA is a critical parameter for the construction of sequencing libraries and the follow-up quantitative analyses. In particular, RNA integrity (RIN) is positively correlated on uniquely mapped reads in RNA-Seq^34^, which means low RIN would lead to a bias in gene expression profiles. In this study, RNA samples with a RIN value >6.5 were employed for RNA-seq library construction, which meant that high-quality reads were obtained for subsequent studies. The quality values for RNA samples, including RIN, are listed in Table 2.

### Quality validation

**Figure 3:**
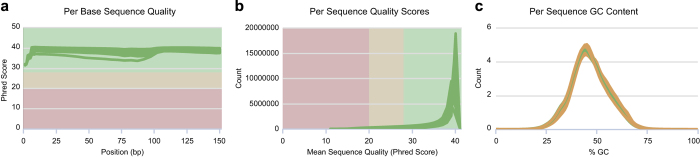
Quality assessment metrics for RNA-seq data. (**a**) Per base sequence quality. (**b**) Per sequence quality scores. (**c**) Per sequence GC content.

**Figure 4:**
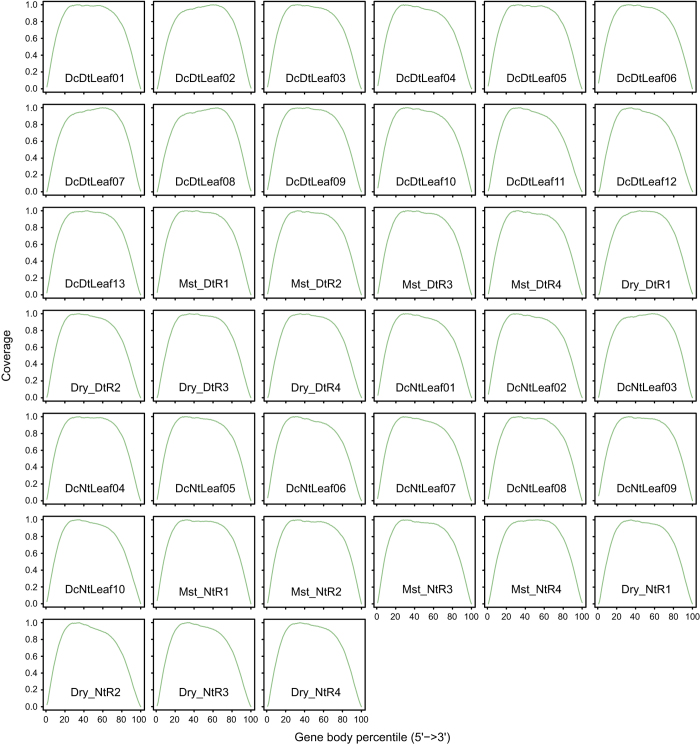
Read distribution on the reference genes. Read distributions are shown for a relative length of 100 reads that were transformed from all reference genes.

The high clean data rate (Table 1), ranging from 98.73% to 99.56%, indicated that both RNA-seq libraries and raw RNA-seq data obtained in this study were of high quality. Results of clean reads assessment by FastQC are illustrated in Fig. 3. The per base quality scores were >30, and most per sequence quality scores were >20, suggesting a high sequence quality. The per sequence GC contents had pattern curves similar to a normal distribution indicating the sequencing data were free of contamination. In addition, we examined read-mapping qualities of the 39 samples, including mapping rates and read distribution on reference genes. The mapping rates to the reference genome were superior, with a range from 83.36% to 88.02% (Table 1). The distribution of reads based on the detection of read coverage skewness showed good fragmentation randomness (Fig. 4), which reflected that each part of the gene was sequenced evenly.

**Figure 5:**
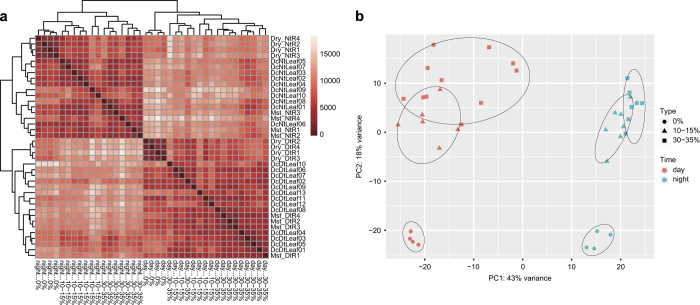
Summary of sample clustering. (**a**) Heatmap displaying similarities among samples based on Poisson distances. (**b**) Principal component analysis performed on the 39 samples based on gene expression profiles.

Both the heatmap (Fig. 5a) and PCA (Fig. 5b) of gene profiles from all 39 samples revealed the clustering of samples according to time and drought level. The samples from daytime and nighttime clustered into two separate groups. The extreme drought groups during both day and night were distinctly separate from the groups with water content of 10–15% and 25–30%. However, the clustering of samples with 10–15% and 25–30% water content overlapped. The explanation for this is that, for a CAM plant, moderate drought would not result in a significant change in gene expression because of its strong ability to adapt to drought.

## Additional information

**How to cite this article**: Wan, X. *et al*. Transcriptomic profiling for prolonged drought in *Dendrobium catenatum*. *Sci. Data*. 5:180233 doi: 10.1038/sdata.2018.233 (2018).

**Publisher’s note**: Springer Nature remains neutral with regard to jurisdictional claims in published maps and institutional affiliations.

## Supplementary Material



## Figures and Tables

**Table 1:** Statistics of Dendrobium catenatum transcriptomes in this study.

Sample	Sampling time	Volumetric water content (%)	Raw reads	Clean reads	Clean read rate (%)	Mapping rate (%)	Index	Biosample accession
Mst_DtR1	Day	30–35	51885148	51634314	99.52	86.58%	ATTGGCTC	SAMN08512106
Mst_DtR2	Day	30–35	50270582	50046482	99.55	87.04%	TTCACGCA	SAMN08512107
Mst_DtR3	Day	30–35	49189742	48998158	99.61	83.36%	GAACAGGC	SAMN08512108
Mst_DtR4	Day	30–35	47456616	47248316	99.56	87.17%	AACTCACC	SAMN08512109
DcDtLeaf01	Day	30–35	55374164	54858496	99.07	87.52%	ATAGCGAC	SAMN09269388
DcDtLeaf02	Day	30–35	57115882	56522666	98.96	86.91%	ATCATTCC	SAMN09269389
DcDtLeaf03	Day	30–35	61685590	61373082	99.49	87.38%	CAAGGAGC	SAMN09269390
DcDtLeaf04	Day	30–35	52698250	52239826	99.13	86.62%	CACCTTAC	SAMN09269391
DcDtLeaf05	Day	30–35	50751892	50514786	99.53	86.70%	CCATCCTC	SAMN09269392
DcDtLeaf06	Day	10–15	50114616	49725348	99.22	86.46%	AATCCGTC	SAMN09269393
DcDtLeaf07	Day	10–15	49147936	48920764	99.54	86.91%	AATGTTGC	SAMN09269394
DcDtLeaf08	Day	30–35	53593676	53296258	99.45	86.27%	AGATGTAC	SAMN09269395
DcDtLeaf09	Day	10–15	55682550	55185044	99.11	86.43%	ACACGACC	SAMN09269396
DcDtLeaf10	Day	30–35	52082812	51800148	99.46	87.11%	TGGTGGTA	SAMN09269397
DcDtLeaf11	Day	10–15	46395690	45908228	98.95	87.92%	CTCAATGA	SAMN09269398
DcDtLeaf12	Day	10–15	46107840	45613946	98.93	88.02%	TGGTGGTA	SAMN09269399
DcDtLeaf13	Day	10–15	54941490	54651394	99.47	86.69%	ACAGATTC	SAMN09269400
Dry_DtR1	Day	0	56670696	56031808	98.87	87.13%	CTGAGCCA	SAMN08512102
Dry_DtR2	Day	0	57586360	57073080	99.11	86.18%	CAATGGAA	SAMN08512103
Dry_DtR3	Day	0	41435504	40966806	98.87	86.92%	GTACGCAA	SAMN08512104
Dry_DtR4	Day	0	42909874	42672078	99.45	86.80%	TTCACGCA	SAMN08512105
Mst_NtR1	Night	30–35	58580260	58285144	99.50	86.72%	AGCACCTC	SAMN08512114
Mst_NtR2	Night	30–35	52135730	51631616	99.03	86.51%	AGCCATGC	SAMN08512115
Mst_NtR3	Night	30–35	46915664	46706968	99.56	85.44%	GAGTTAGC	SAMN08512116
Mst_NtR4	Night	30–35	52966336	52700452	99.50	86.84%	CCTCTATC	SAMN08512117
DcNtLeaf01	Night	30–35	53175526	52912342	99.51	86.81%	TGGAACAA	SAMN09269401
DcNtLeaf02	Night	10–15	53372658	53101428	99.49	85.11%	CTAAGGTC	SAMN09269402
DcNtLeaf03	Night	10–15	54473652	54026066	99.18	86.63%	CGACACAC	SAMN09269403
DcNtLeaf04	Night	10–15	51474354	51206284	99.48	85.30%	CGGATTGC	SAMN09269404
DcNtLeaf05	Night	10–15	56221144	55922546	99.47	86.42%	CCGACAAC	SAMN09269405
DcNtLeaf06	Night	30–35	52075438	51809058	99.49	86.19%	GACAGTGC	SAMN09269406
DcNtLeaf07	Night	10–15	54910042	54598814	99.43	86.60%	CCTAATCC	SAMN09269407
DcNtLeaf08	Night	30–35	50011312	49756262	99.49	86.83%	TGGCTTCA	SAMN09269408
DcNtLeaf09	Night	10–15	52772882	52521906	99.52	87.49%	AAGAGATC	SAMN09269409
DcNtLeaf10	Night	10–15	56806334	56179634	98.90	86.89%	GATGAA & GATGAATC	SAMN09269410
Dry_NtR1	Night	0	41989278	41591930	99.05	87.90%	CATCAAGT	SAMN08512110
Dry_NtR2	Night	0	41976282	41462370	98.78	86.63%	CTAAGGTC	SAMN08512111
Dry_NtR3	Night	0	55341820	55035648	99.45	86.60%	AGGCTA & AGGCTAAC	SAMN08512112
Dry_NtR4	Night	0	44553838	44105524	98.99	87.21%	ACCTCCAA	SAMN08512113
Clean data rate=Clean read number/Raw read number ^∗^ 100%. Mapping rates were assessed from the Hisat2 mapping procedure.								

**Table 2:** RNA sample quality for each sample.

Sample	RIN	25S/18S	OD260/280	OD260/230
Mst_DtR1	7.2	1.8	1.9	2.4
Mst_DtR2	7.4	1.3	1.9	2.5
Mst_DtR3	7.1	1.6	2.0	2.5
Mst_DtR4	7.5	2.1	1.9	2.5
DcDtLeaf01	7.7	2.0	1.8	2.2
DcDtLeaf02	7.2	1.9	1.8	2.2
DcDtLeaf03	7.5	1.8	2.0	2.1
DcDtLeaf04	7.5	2.0	2.0	2.3
DcDtLeaf05	7.4	2.3	1.9	2.3
DcDtLeaf06	7.8	2.4	1.8	2.3
DcDtLeaf07	7.7	2.1	2.0	1.6
DcDtLeaf08	7.8	4.5	1.7	1.8
DcDtLeaf09	7.5	1.8	2.0	2.0
DcDtLeaf10	7.8	1.7	2.0	2.4
DcDtLeaf11	8.5	1.7	2.0	3.0
DcDtLeaf12	7.5	1.3	2.1	3.3
DcDtLeaf13	6.9	1.6	1.9	2.4
Dry_DtR1	8.0	1.7	2.0	2.5
Dry_DtR2	6.8	1.8	2.4	3.4
Dry_DtR3	6.5	1.2	2.1	2.9
Dry_DtR4	7.1	1.6	2.0	2.6
Mst_NtR1	7.2	1.6	2.1	2.6
Mst_NtR2	7.9	1.9	2.1	2.4
Mst_NtR3	7.2	1.7	2.1	2.3
Mst_NtR4	7.7	2.0	2.0	2.5
DcNtLeaf01	7.7	1.6	2.1	2.6
DcNtLeaf02	7.4	2.0	1.9	2.4
DcNtLeaf03	7.1	2.1	1.5	2.4
DcNtLeaf04	6.6	1.6	2.0	2.6
DcNtLeaf05	7.7	2.0	1.9	2.3
DcNtLeaf06	7.2	1.6	2.0	2.3
DcNtLeaf07	6.5	1.6	2.0	2.1
DcNtLeaf08	7.5	2.0	1.8	2.4
DcNtLeaf09	7.5	2.7	1.7	2.7
DcNtLeaf10	8.3	1.6	2.2	3.7
Dry_NtR1	7.7	1.7	2.0	2.6
Dry_NtR2	8.1	1.7	2.0	2.4
Dry_NtR3	7.9	1.6	2.0	2.5
Dry_NtR4	8.0	1.7	2.1	2.9
